# Altered Default Mode Network and Salience Network Functional Connectivity in Patients with Generalized Anxiety Disorders: An ICA-Based Resting-State fMRI Study

**DOI:** 10.1155/2020/4048916

**Published:** 2020-08-14

**Authors:** Hang Xiong, Rong-Juan Guo, Hua-Wei Shi

**Affiliations:** ^1^Department of Medical Neurology, Tongzhou District of Dongzhimen Hospital Affiliated to Beijing University of Chinese Medicine, Beijing 100001, China; ^2^Department of Medical Neurology, Dongfang Hospital Affiliated to Beijing University of Chinese Medicine, Beijing 100006, China; ^3^Departmen of Medical Neurology, Dongzhimen Hospital Affiliated to Beijing University of Chinese Medicine, Beijing 10001, China

## Abstract

This study aimed to explore the role of the default mode network (DMN) and salience network (SN) in the assessment of pathophysiology of generalized anxiety disorder (GAD) through analyzing the characteristics of internal function connectivity (FC) and to investigate the relationship of FC with Hamilton anxiety (HAMA) scale scores in untreated GAD patients during a resting-state functional magnetic resonance imaging (rs-fMRI). Rs-fMRI and HAMA scale scoring were performed in 51 GAD patients (31 GAD patients with liver stagnation transforming into fire type and 20 GAD patients with stagnation of liver-Qi syndrome type) and 20 healthy controls. Spearman correlation analysis was performed to assess the association between HAMA scores and abnormal brain FC. Compared with healthy controls, the FC of the right medial prefrontal gyrus of the DMN and the right superior temporal gyrus of the SN increased significantly in the GAD patients (*P* < 0.001). However, the FC of the left middle frontal gyrus and bilateral medial superior frontal gyrus of the SN reduced significantly in the GAD patients with stagnation of liver-Qi syndrome type as compared with healthy controls and GAD patients with liver stagnation transforming into fire type (*P* < 0.001). There was no relationship between abnormal brain FC and HAMA scores. In conclusion, the FC of the DMN and SN may be abnormal in the GAD patients at the resting state. The aberrant FC of some crucial brain regions of these networks may contribute to the pathophysiology of GAD.

## 1. Introduction

Generalized anxiety disorder (GAD) is characterized by persistent and excessive worry about a number of different things. It is hard to control, most of the causes originate from the family, health, finance, and other problems, and it is usually accompanied by other nonspecific psychological and physiological symptoms [1]. The main characteristics of GAD are chronic excessive anxiety and worry [2], which significantly reduce the work efficiency and increase the risk for other diseases. Among anxiety disorders, GAD has the highest prevalence and affects 4–6% of the general population [3].

Traditional Chinese medicine (TCM) is a kind of complementary and alternative medicine, which has unique theory and abundant preventative and therapeutic methods [4]. Syndrome differentiation is one of the basic features of TCM, and the clinical treatment is usually based on the TCM syndrome which is a diagnostic classification of pathological changes of disease state according to the individual symptoms and signs, such as pulse and tongue. Thus, TCM emphasizes the role of individual characteristics in clinical diagnosis and treatment [5]. Anxiety disorders are mainly caused by emotional discomfort, depression, and Qi stagnation according to TCM theory. The basic pathogenesis of GAD is that the liver fails to maintain the normal flow of Qi, resulting in the stagnation of liver Qi, and the basic rule for its treatment is to regulate Qi-movement. However, “fire” symptoms will be turned out after long-term liver-Qi stagnation. In the TCM, strong people tend to have more fire, while people with poor physique tend to be in a liver-Qi stagnation state. In addition, people with poor physique often have a longer course of disease. In fact, the stagnation of liver-Qi and stagnation of Qi are crucial for the onset of this disease [6]. Thus, in TCM, the stagnation of the liver-Qi syndrome type and liver stagnation transforming into the fire type are common. In our previous study, results showed that Ningxin Anshen formula was effective to improve the clinical symptoms of mild-to-moderate GAD, with the effectiveness rate of as high as 83.33% [7].

Although GAD is a common anxiety disorder, few studies have been conducted to investigate its neurophysiology. At present, the diagnosis of GAD mainly depends on the clinical symptoms and signs. Therefore, to reveal the pathophysiology of GAD and find relatively objective biomarkers of GAD are of great significance.

The magnetic resonance imaging (MRI) can noninvasively assess the state of brain connectivity and determine whether it is dynamic (functional connectivity) or structural (structural connectivity). The resting-state functional magnetic resonance imaging (rs-fMRI) can show the function and structure of the brain by assessing the signal changes in brain blood-oxygen at baseline, which reflects the real-time local activity of brain tissues [8]. In recent years, rs-fMRI has been widely used to assess the brain dysfunction in various mental disorders. Some studies have shown that the resting-state whole brain functional networks are related to anxiety [9, 10]. The default mode network (DMN) includes the medial prefrontal lobe, posterior cingulate gyrus, and other brain areas, and these brain regions are more active at the resting state than other brain networks. DMN is usually described as a unified, homogeneous system, mainly involving the integration of autobiographical memories, self-monitoring, and retrieval and manipulation of past events to solve problems and formulate future plans, as well as emotional regulation [11]. It has been proposed that the DMN plays a key role in orchestrating the cognitive functions such as introspection, prospective memory, and a variety of processes described intuitively as daydreaming or mind-wandering [12, 13]. The DMN dysfunction has also been implicated in some pathological states including autism [14], schizophrenia [15], and depression [16]. This abnormality may be the basis of self-information processing and emotional processing bias in the GAD [17]. The resting-state functional connectivity patterns in DMN were compared between GAD patients and nonanxious controls, and results showed that the functional connectivity between the posterior cingulate gyrus and the medial prefrontal cortex in the elderly was more affected by anxiety than in the young population [18]. In addition, the effects of clinical anxiety on the prefrontal lobe and marginal junction have been assessed by the structure, function (i.e., resting state), and MRI [19]. There is a difference in the resting-state connectivity between the posterior hippocampal connectivity with the default mode network and the anterior hippocampal connectivity to the limbic-prefrontal circuitry among healthy subjects, posttraumatic stress disorder patients, and GAD patients [20].

In contrast to the DMN, the human salience network (SN) is a distributed set of cortical and subcortical regions involved in detection and response to highly relevant (salient) stimuli [21, 22]. The SN was first identified using conventional fMRI to measure the blood oxygen level-dependent (BOLD) responses during a spatial working memory task in patients [22]. In this task, several cortical regions (including the frontoinsular cortex) display significant BOLD responses. The insular cortex is subsequently used as a “seed region” for an independent component analysis of its functional connectivity patterns. The known functions and modalities processed in these brain regions include attention, sensation, visceral activity, affection, and limbic system, and thus, investigators speculate that this network processes perceptual salience and thus termed it the “salience network” [23]. The discovery of the SN also reveals an interesting clinical relationship: the strength of functional connectivity within the SN is strongly related to the visual analog scores from a prescan anxiety assessment. This correlation suggests a tight link between the strength of the SN and states of vigilance [23]. These findings are also confirmed by recent studies. In addition, studies also indicate that the functional connectivity within the SN is aberrant in many neurological disorders including anxiety [24], posttraumatic stress disorder [25], depression [21], psychosis with auditory delusions [26], and affective disorders [27].

However, a more detailed examination of these anatomical positions and features will be helpful for understanding the neural underpinnings of GAD [28]. Therefore, the study of functional connectivity between two networks may help us understand the role of the networks in the pathogenesis of GAD. This study was performed to assess the alterations in the DMN and SN in GAD patients (including stagnation of the liver-Qi syndrome group and the liver stagnation transforming into fire group) and healthy controls by using rs-fMRI and to evaluate the relationship of specific changes in the DMN and SN with the HAMA scale scores in GAD patients.

## 2. Materials and Methods

### 2.1. Participants

GAD patients were recruited from the Department of Medical Neurology at Dongzhimen Hospital of Beijing University of Chinese Medicine between November 2015 and June 2017. The GAD was diagnosed by two experienced psychiatrists. The inclusion criteria were as follows: (1) GAD was diagnosed according to the diagnostic criteria from the *American Diagnostic and Statistical Manual of Mental Disorders*, 5th ed. [29]; (2) patients complained of uncontrollable anxiety and worries about everyday events and problems for at least 6 months; (3) patients were 20–40 years old; (4) all the participants were right-handed; and (5) GAD patients had the HAMA score higher than 14.

GAD subtyping was done according to the *Guidelines for Diagnosis and Treatment of Common Diseases in Internal Medicine of Traditional Chinese Medicine* [30]. Thirty-one GAD patients with liver stagnation transforming into fire type (mean age, 30.7 ± 4.7 years) and twenty GAD patients with stagnation of liver-Qi syndrome type (mean age, 30.9 ± 3.8 years), and twenty age-, sex-, hand-, and education-matched healthy controls (mean age, 29.4 ± 5.9 years) were recruited in the present study. Volunteers with a HAMA score less than 7 were included as controls (Table 1). All patients were diagnosed with primary GAD and not treated before admission.

Exclusion criteria were as follows: (1) patients had a history of neurological or psychiatric disorders or cognitive dysfunction, such as depression, obsessive-compulsive disorder, schizophrenia, PTSD, and bipolar disorder; (2) patients had severe liver, heart, and kidney dysfunction; (3) patients were pregnant or breast-feeding women; (4) patients also had secondary anxiety caused by hyperthyroidism or a history of alcohol or other substance abuse in the year before testing; and (5) patients developed severe diseases that did not allow them to participate in this study [28]. All participants were informed of the safety and eligibility criteria for fMRI scanning. The study was approved by the Research Ethics Committee of Dongzhimen Hospital of Beijing University of Chinese Medicine. All subjects provided written informed consent before study.

### 2.2. Image Acquisition

Resting-state fMRI and structural data were acquired on a 1.5 Tesla MRI scanner (Philips Intera, Philips Medical Systems, Best, the Netherlands) with head coils in Dongzhimen Hospital, Beijing University of Traditional Chinese Medicine. Each subject lied on a foam pad to reduce head motion in a supine position with a headphone to reduce noise. In the data acquisition, all subjects were asked to close their eyes, relax, keep calm, not think about anything systematically, and not fall asleep.

Rs-fMRI data were acquired using a single-shot gradient-echo planar imaging sequence, and the following parameters were used: echo time (TE) = 50 ms, repetition time (TR) = 3000 ms, flip angle (FA) = 90°, matrix = 64 × 64, number of excitations (NEX) = 1, slice thickness = 3.6 mm, slice gap = 0.72 mm, field of view (FOV) = 232 mm × 232 mm, voxel size = 3.63 mm × 3.68 mm × 3.6 mm, and dynamics scans = 60. fMRI scanning for each subject lasted for 6 min and 12 sec. For the T1-weighted structural imaging, a two-dimensional inversion-recovery turbo spin echo (2D-IR-TSE) sequence was used, and the parameters were as follows: TE = 15 ms, TR = 5165 ms, matrix = 384 × 384 × 75%, FA = 90°, number of slices = 33, FOV = 232 mm × 232 mm, voxel size = 0.6 mm × 0.82 mm × 3.6 mm, and total duration = 3.05 min. After the MRI scanning, the patient was assessed if he or she had followed the instructions and remained awake throughout the scanning. Those who did not comply with the instructions were excluded from this study.

### 2.3. Image Processing and Independent Component Analysis

All rs-fMRI data were classified and transformed into the NIFTI format using MRI Convert and then preprocessed using DPABI (a toolbox for Data Processing and Analysis of Brain Imaging; http://www.rfmri.org) [31] software. DPABI was used to process the data in several steps: first, 10 initial time points were removed because of scanning noise and spin saturation, and the remaining time points were used for further analysis. Images were realigned for motion correction and slice timing correction. The data of subjects with excessive head motion (>3 mm in translation or 3° in rotation) were not used for further analysis.

After head motion correction, fMRI images were standardized to the Montreal Institute of Neurology template (resampling voxel size = 3 × 3 × 3 mm^3^). Linear trends and temporal filtering (band pass, 0.01–0.08 Hz) were removed to reduce very low-frequency drift and physiologic high-frequency respiratory and cardiac noise. Then, spatial smoothing was performed on the standardized individual with a Gaussian kernel of 6 mm full-width at half-maximum [32].

Independent component analysis (ICA) is one of the most widely used data-driven methods for the processing of fMR imaging data. It can realize complete data-driven discovery of spatial patterns, temporal covariances, and even groups. ICA is very suitable for fMR imaging analysis because of its robustness to artifacts, minimum assumption of temporal process or spatial pattern shape, and easy estimation. One of the most attractive features of ICA is its ability to extract components from hybrid fMR imaging signals, representing large-scale neural networks [33]. ICA was carried out for fMRI data analysis using the fMRI toolbox, called GIFT (group ICA for fMRI; http://icatb.sourceforge.net/) [34]. Data from each subject were concatenated across time and calculated for subject-specific components using the Infomax algorithm which is based on the principle of maximum information transfer [35]. The independent component number (ICN) was 50. Best-fit DMN and SN components were selected manually using the template in GIFT. Scans with problems related to best-fit DMN and SN component selection were excluded.

### 2.4. Statistical Analysis

SPSS version 20.0 (IBM Corporation, Armonk, NY, USA) was used to analyze demographic data and clinical characteristics. Chi-square test was used to compare the gender. The age, education level, course of disease, and HAMA scores were compared between GAD patients and healthy controls with two-sample *t*-test. A value of *P* < 0.05 was considered statistically significant [32]. Firstly, the DMN and SN were tested with single-sample *t* test and false discovery rate (FDR) correction, respectively, in the liver-Qi stagnation group, liver stagnation transforming into fire group, and health control group (*P* < 0.05). The mask of each network in each group was made, and then the combination of three groups was taken as the statistical range of the network. Subsequently, analysis of variance (ANOVA) was used to compare the functional connectivity among three groups. Permutation test [36] based on the threshold-free cluster enhancement (TFCE) was used to make multiple comparisons and corrections, and significant brain regions with family-wise error (FEW) correction were obtained. Subsequently, the mean value of each brain region with significant difference between groups was extracted for statistical analysis in SPSS. ANOVA was used for comparisons between two groups followed by using post hoc test. Finally, the functional connectivity of the DMN and SN was analyzed among 3 groups. Pearson's correlation analysis with 95% confidence interval (CI) was used to analyze the correlation of functional connectivity of the DMN and SN with significant differences with HAMA scores.

## 3. Results

### 3.1. Demographic and Clinical Characteristics

There were no significant differences in the gender (*P*=0.50), age (*P*=0.32), and education level (*P*=0.28) between the GAD group and the healthy control group. The HAMA scores in the GAD group were significantly higher than those in the healthy control group. The demographic and clinical characteristics are shown in Table 1 [32].

### 3.2. Networks Extracted by ICA

**Figure 1:**
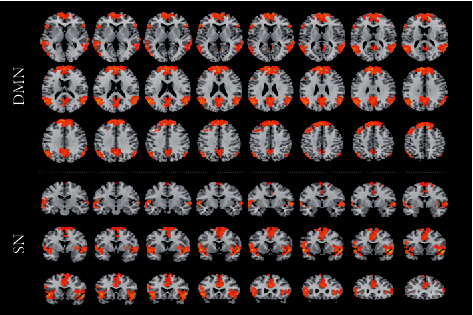
DMN and SN. Voxel intensities represent the connectivity and degree of coactivation in the resting-state network. Voxels which were negatively associated with the network were excluded.

The DMN and SN extracted by ICA are shown in Figure 1. DMN mainly included the bilateral medial prefrontal lobe, posterior cingulate/anterior cuneate lobe, angular gyrus, and other brain regions. The SN mainly included the anterior cingulate gyrus, anterior insula, and other brain regions.

### 3.3. Functional Connectivity Analysis in the DMN

**Figure 2:**
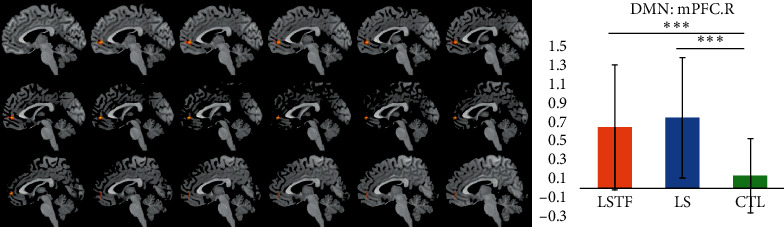
Brain regions with significant difference in the functional connectivity of mPFC.R (DMN) in the LSTF group, LS group, and control group.

Compared to the baseline (ANOVA), GAD patients showed significantly increased connectivity of the right medial prefrontal gyrus (mPFC.R) (Figure 2 and Table 2). The brain regions with significant difference were extracted, and AVONA confirmed significant differences in this brain region (*F* (2, 68) = 16.717, *P* < 0.001, Table 3). Post hoc test showed mPFC.R in the liver-Qi stagnation group (group LS) and the liver stagnation transforming into fire group (group LSTF) was significantly different from that in the healthy control group (*P* < 0.001), while there was no significant difference between the LS group and the LSTF group (*P*=0.821, Figure 2).

### 3.4. Functional Connectivity Analysis in the SN

**Figure 3:**
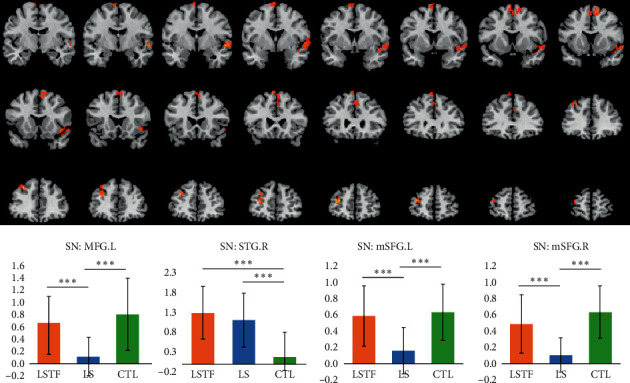
Brain regions with significant difference in the functional connectivity of the SN in the LSTF group, LS group, and control group.

Compared to the baseline (ANOVA), GAD patients showed significantly decreased connectivity of the bilateral medial superior frontal gyrus (mSFG.L, mSFG.R, with supplementary motor areas) and left middle frontal gyrus (MFG.L), while the connectivity of the right superior temporal gyrus (STG.R) significantly increased (Figure 3 and Table 2). The brain regions with significant difference were extracted, and AVONA confirmed significant differences in these brain regions (Table 3; *P* < 0.001). Post hoc test revealed GAD patients showed significantly increased connectivity of STG.R as compared with the control group (*P* < 0.001), while there was no significant difference between the LS group and the LSTF group (*P*=0.621, Figure 3). LS group showed lower connectivity of the MFG.L, mSFG.L, and mSFG.R as compared with the control group and LSTF group (*P* < 0.001), while there was no significant difference between the control group and the LSTF group (*P*=0.387, *P*=0.886, and *P*=0.795, Figure 3).

### 3.5. Correlation between Functional Connectivity and HAMA Scores

The extracted FC values of the DMN and SN in GAD patients had no relationship with the HAMA scores as shown in the Pearson correlation analysis (Table 4).

## 4. Discussion

In the resting state (i.e., no task), the brain displays spontaneous, low-frequency neuronal oscillations and has spatiotemporal correlation, which defines a large-scale, functional connection network. These resting-state networks can be indirectly measured by resting-state or task-free fMRI. rsfMRI has been widely used in the neuroscience field because it is not affected by the patient's executive power, is easy to operate, and has good repeatability. ICA is the most widely used mathematical technique that can decompose the fMRI data set into independent components and separate noise into distinct components, which avoids the need for regressing out these signals during preprocessing [37].

This study showed abnormal functional connectivity in the right medial prefrontal lobe (mPFC.R) of the DMN. The prefrontal lobe is the high-level center of emotion regulation and can inhibit the behavioral response to fear. The functional abnormality of the prefrontal lobe may be related to the pathogenesis of GAD [38, 39]. Liao et al. found that the functional connection of mPFC in the DMN was enhanced in the social anxiety disorder [40]. In addition, mPFC is the prominent region with higher sensitivity in the identification of adolescent GAD [41]. The abnormal FC dynamics associated with mPFC revealed the disrupted information exchange related to this region and might result in the failure to sense “self- and others' thinking, feeling, perceiving, imagining, reacting, attributing, and inferring,” [42] which made the GAD patients unable to control their worry and anxiety as sensitively as healthy controls. In a word, the above findings support the hypothesis of compensatory activation of the prefrontal lobe in the theory of emotional disorder [43]. Post hoc test showed that the LS group and the LSTF group were significantly different from the control group (*P* < 0.001) in the brain functional connectivity of the right medial prefrontal gyrus of the DMN, while there was no significant difference between the LS group and the LSTF group. This means there is little difference in the brain functional connectivity of the right medial prefrontal gyrus of the DMN between the liver-Qi stagnation group and the liver stagnation transforming into fire group. Our results provide additional evidence on the notion that DMN plays a key role in the pathophysiology of GAD.

As compared to the baseline, GAD patients had significantly different connectivity of the bilateral medial superior frontal gyrus (mSFG.L, mSFG.R, with supplementary motor areas), MFG.L, and STG.R of the SN. The superior frontal gyrus (SFG) is located at the superior part of the prefrontal cortex and involved in the cognition and motor controls. Specifically, the SFG including the supplementary motor area (SMA) is mainly activated by motor tasks [44]. Some GAD patients show excessive tension, leading to inability to sit still or other somatic symptoms. Our results indicated the functional activity of GAD patients decreased in the mSFG, suggesting that some symptoms of GAD patients may be related to the abnormality of some brain areas related to motor control. As compared to controls, GAD patients showed decreased functional connectivity of MFG.L, which belongs to the subregion of the frontal lobe. These results suggest the prefrontal cortex of GAD patients is insufficient to inhibit the muscle tension and motor disturbance. Post hoc test revealed that the LS group showed significantly lower connectivity of the MFG.L and mSFG as compared with the control group and LSTF group (*P* < 0.001), while there was no significant difference between the control group and the LSTF group. This indicates that there is significant difference in both MFG.L and mSFG of the SN between the liver-Qi stagnation group and the liver stagnation transforming into fire group. In the present study, our results showed the STG.R was related to the “liver” in the TCM. In the TCM, stagnation of liver-Qi syndrome is the most early characteristic of liver failure to maintain the normal flow of Qi, and thus, the functional abnormality of the left middle frontal gyrus and bilateral medial superior frontal gyrus occurs earlier as compared with the right superior temporal gyrus. Studies have shown that the right STG is the key node involved in self- and theory of mind-processing [45]. In addition, patients with GAD showed a significant reduction in the gray matter volumes, especially in the regions of the superior temporal gyrus, compared with healthy controls [46]. These may be responsible for the hepatic dysregulation in the TCM. To date, few studies have been undertaken to investigate the relationship of the middle frontal gyrus and superior frontal gyrus with GAD. More studies with large sample size are needed to confirm our findings.

Post hoc test also indicated that GAD patients had significantly increased connectivity of STG.R as compared with the control group (*P* < 0.001) in the SN. Superior temporal gyrus (STG) is an important temporal area involved in the insight and mainly located above the lateral temporal lobe [47]. It has been reported that a key cognitive process for distinguishing insight solutions from noninsight solutions is that insightful solutions require the solver to identify distant or novel semantic (or associative) relations. Therefore, insight into specific neurological activities should reflect this process. The area that is most likely to contribute to insight into problem-solving is the STG.R. Generally speaking, semantic integration is important for connecting various problem elements together and connecting the problem to the solution, and the coarse-coded semantic integration using STG.R computing is particularly important for insights into solutions, at least for verbal problems (or problems that can be solved with verbal or semantic information) [48]. In order to elucidate the brain mechanisms underlying the process of chunk decomposition, Luo et al. [49] developed a task that uses Chinese characters as materials. Chinese characters are ideal examples of perceptual chunks. During the executive processes contributing to chunk decomposition, stronger activations in the STG.R were confirmed. This indicated that the activation of the STG.R was mainly related to the active construction and formation of novel and long-distance associations [47]. Liu et al. [50] found an increased functional connectivity of the STG. Thus, abnormalities in this region may lead to symptoms of excessive anxiety in GAD patients.

This was the first neuroimaging study focusing on the brain activity in GAD patients with different TCM syndromes. Our results confirmed the abnormal brain function of GAD patients and preliminarily indicated that the change in cerebral activity was also different in GAD patients with different TCM syndromes. There were still limitations in this study. Firstly, there were many subtypes according to the TCM classification of GAD [51], but only two of the most common GAD subtypes were studied. Whether other subtypes are different from the subtypes investigated in this study is also needed to be further investigated. Secondly, TCM diagnostic procedures are mainly based on the experience and subjective assessments. Perhaps more objective diagnostic methods of TCM are needed for the assessment. Thirdly, the sample size of our study was relatively small, and thus, applicability of our findings may therefore be limited. In addition, different responses of two subtypes to anxiolytic treatment were not assessed in our study [52]. Moreover, patients with severe GAD based on HAMA scores were not enrolled in our study. Further investigations are needed to highlight the relationship between the network and the central executive network in GAD patients and to confirm our findings in a larger population [53].

## 5. Conclusions

Our study investigated the brain functional connectivity in patients with different subtypes of GAD based on syndrome differentiation in the TCM and the healthy controls, and our results confirmed that GAD patients had cerebral dysfunction at the resting state. Moreover, the brain activity of GAD patients with liver-Qi stagnation type was different from that of patients with liver stagnation transforming into fire type in the SN at the resting state. Our study provides an objective basis for the differentiation of GAD subtypes and a new way for further investigation of the mechanism of TCM typing. In general, our study may improve our understanding of the neural underpinnings of GAD and is also helpful for the identification of etiology of GAD, which is beneficial for the determination of specific treatment. Resting-state design is an important field of neuropsychology in the future, which investigates the relationship among diseases, symptom characteristics, potential network abnormalities, and severity of symptoms [54].

## Figures and Tables

**Table 1:** Characteristics of GAD patients and healthy controls (*x* ± *s*).

	Liver stagnation transforming into fire type (*n* = 31)	Stagnation of liver-Qi syndrome type (*n* = 20)	Healthy controls (*n* = 20)
Men/women	15/16	10/10	10/10
Age (years)	30.7 ± 4.7	30.9 ± 3.8	29.4 ± 5.9
Education level (years)	16.3 ± 2.6	16.6 ± 2.4	16.5 ± 3.3
Cause of disease (months)	8.7 ± 2.1	8.8 ± 2.6	0
HAMA scale score	18.17 ± 2.52	17.87 ± 2.00	1.20 ± 0.90

**Table 2:** Brain regions with significant differences in the DMN and SN.

	Brain region	Peak *F*-value	Voxel	Peak MNI coordinates		
				*x*	*y*	*z*
DMN						
1	mPFC.R (right medial prefrontal gyrus)	17.228	27	0	51	0
SN						
1	STG.R (right superior temporal gyrus)	13.049	127	60	−3	−3
2	MFG.L (left middle frontal gyrus)	18.956	70	−30	51	15
3	mSFG.L (left medial superior frontal gyrus)	11.085	81	−3	0	78
4	mSFG.R (right medial superior frontal gyrus)	10.351	63	9	13	59

*Note.* MNI coordinates: a three-dimensional human brain coordinate-positioning system created by Montreal Neurological Institute (MNI).

**Table 3:** Brain regions with significant differences among three groups.

Network	Brain region	Functional connectivity (*X* ± SD)			ANOVA	
		LSTF group	LS group	Control group	*F* (2, 68)	*P*
DMN						
1	MFC.R	0.649 ± 0.665	0.751 ± 0.641	0.133 ± 0.393	16.717	<0.001
SN						
1	STG.R	1.296 ± 0.659	1.122 ± 0.675	0.195 ± 0.622	18.370	<0.001
2	MFG.L	0.628 ± 0.469	0.113 ± 0.322	0.807 ± 0.591	19.754	<0.001
3	mSFG.L	0.584 ± 0.369	0.155 ± 0.285	0.630 ± 0.344	14.504	<0.001
4	mSFG.R	0.484 ± 0.355	0.105 ± 0.212	0.630 ± 0.321	12.036	<0.001

*Note.* mPFC.R: right medial prefrontal gyrus; STG.R: right superior temporal gyrus; MFG.L: left middle frontal gyrus; mSFG.L: left medial superior frontal gyrus; mSFG.R: right medial superior frontal gyrus; and FNC: functional network connectivity.

**Table 4:** Correlations of HAMA scores with brain regions with significant differences in the functional connectivity of the DMN and SN.

	Brain region	Statistics	
		*r*	*P*
DMN			
1	MFC.R	0.419	0.228
SN			
1	STG.R	0.154	0.671
2	MFG.L	−0.197	0.586
3	mSFG.L	−0.304	0.392
4	mSFG.R	−0.492	0.149

## Data Availability

The data used to support the findings of this study are available from the corresponding author upon request.
